# Primary *Pneumocystis* Infection in Infants Hospitalized with Acute Respiratory Tract Infection

**DOI:** 10.3201/eid1301.060315

**Published:** 2007-01

**Authors:** Hans Henrik Larsen, Marie-Louise von Linstow, Bettina Lundgren, Birthe Høgh, Henrik Westh, Jens D. Lundgren

**Affiliations:** * Hvidovre University Hospital, Copenhagen, Denmark

**Keywords:** Pneumocystis, respiratory tract infection, infant, child, PCR, research

## Abstract

Acquisition of *Pneumocystis jirovecii* infection early in life has been confirmed by serologic studies. However, no evidence of clinical illness correlated with the primary infection has been found in immunocompetent children. We analyzed 458 nasopharyngeal aspirates from 422 patients hospitalized with 431 episodes of acute respiratory tract infection (RTI) by using a real-time PCR assay. In 68 episodes in 67 infants, *P. jirovecii* was identified. The odds ratio (95% confidence interval) of a positive signal compared with the first quartile of age (7–49 days) was 47.4 (11.0–203), 8.7 (1.9–39.7), and 0.6 (0.1–6.7) for infants in the second (50–112 days), third (113–265 days), and fourth (268–4,430 days) age quartiles, respectively. Infants with an episode of upper RTI (URTI) were 2.0 (1.05–3.82) times more likely to harbor *P. jirovecii* than infants with a lower RTI. *P. jirovecii* may manifest itself as a self-limiting URTI in infants, predominantly those 1.5–4 months of age.

## Abstract 2

Primary *P. jirovecii* infection may appear as a self-limiting upper respiratory tract infection in infants.

The opportunistic fungus *Pneumocystis jirovecii* (formerly *Pneumocystis carinii* f.sp. *hominis* [*1*]) may cause severe pneumonia (PCP) in patients with AIDS and other immunodeficiencies. The epidemiology of *P. jirovecii* infection is still not well understood, however. Serologic studies have shown that children are exposed to *P. jirovecii* early in life (*2*–*5*).

To our knowledge, no previous evidence exists of a correlation between clinical illness and primary infection in the competent host (*6*). Recently, *P. jirovecii* has been found in respiratory secretions from infants with respiratory tract infection (RTI) as well as in autopsy lung tissue from infants who died of sudden infant death syndrome (*7*–*10*).

The role of a human reservoir for the pathogen, consisting of HIV-positive or HIV-negative adults, has recently been debated (*11*). Also, immunocompetent children may contribute to the circulation of the organism. In addition, detecting abundant infection in infants could reflect widespread exposure from an environmental reservoir.

We conducted a blinded, retrospective study to determine the prevalence of *P. jirovecii* harbored in the respiratory tracts of Danish children with acute RTI, and whether clinical and laboratory characteristics separate those with and without *P. jirovecii* infection*.* The detection method employed was a single-round, closed-tube, real-time PCR assay. The study was approved by the Ethical Committee of Copenhagen (KF 01–028/03).

## Methods

### Patient Population and Samples

All available routine nasopharyngeal aspirates (NPAs) obtained for respiratory syncytial virus (RSV) analysis during 1999–2002 from children hospitalized at the Departments of Pediatrics, Hvidovre University Hospital and Amager Hospital, Copenhagen, Denmark, were included in the study. Thus, included samples and subjects were NPAs from children in whom the treating physician suspected or wished to rule out an RSV infection. Therefore, most children were <24 months of age. Samples collected within 3 weeks from the same person were regarded as being from the same episode of respiratory disease.

### Clinical Data Collection

Clinical data were obtained by reviewing medical records of the patients using uniform data abstraction forms. The reviewer, a pediatrician, was blinded to PCR data. A diagnosis of lower RTI (LRTI), upper RTI (URTI), or “other” was made on the basis of recorded clinical findings (*12*). In brief, a diagnosis of URTI was made if the infant had one or more of the following clinical signs without evidence of LRTI: cough, nasal discharge, a red bulging tympanic membrane, and pharyngotonsillar erythema or exudate. A diagnosis of LRTI was made if the infant also had abnormal sounds on lung auscultation and chest indrawing or tachypnea. Infants with an RTI who were hospitalized primarily for other reasons received the diagnosis “other.”

### PCR Analysis

All samples were extracted and assayed in the Clinical Microbiology Laboratory at Hvidovre University Hospital. Universal PCR laboratory procedures were used, such as physical separation of the steps involved in PCR and unidirectional workflow; specimens were processed carefully with observance of universal PCR laboratory precautions. In addition, a single-round, nonnested, closed-tube PCR assay, with no manipulations of amplicons required, inherently reduces the risks of carryover contamination. To further reduce this risk, uracil-N-glycosylase and deoxyuridine triphosphate were used to prevent amplicon carryover (*13*). PCR analysis was carried out with researchers blinded to all clinical data. The code was only broken at the time PCR analysis and clinical data collection were completed.

### DNA Extraction

DNA was extracted from patient and control specimens with the automated MagnaPure (Roche Diagnostics GmbH, Mannheim, Germany) system, using the MagNA Pure LC DNA Kit III (bacteria, fungi) (Roche), according to the manufacturer’s recommendations. A sample volume of 100 μL was used for extraction, and a preincubation step was carried out by adding lysis buffer and protein K mixture to the sample, which was then incubated for 15 min at 65°C, followed by 10 min at 95°C. Extracted material was stored at −80°C.

### PCR Controls

*P. jirovecii*–positive and –negative respiratory samples, as determined by results of previous microscopy and PCR, were included with each DNA extraction and in each PCR run as external controls. An internal control amplifiable by the *P. jirovecii* primers was included to detect PCR inhibitors in the patient specimens (*14*).

The following standards were set. For *Pneumocystis*, 10-fold serial dilutions (10^−2^–10^5^ copies/μL) of a plasmid containing a *P. jirovecii* major surface glycoprotein (MSG) gene insert were prepared (*14*). Standard curves for quantification of positive patient samples were generated by assaying the serial dilution in triplicate. For betaglobin, 10**-**fold serial dilutions (1.5 × 10^−4^–1.5 × 10^1^ ng/μL]) of human genomic DNA provided with the Control Kit DNA (Roche) were used to generate standard curves, according to the manufacturer’s instructions.

DNA amplification and detection were carried out as follows. For *Pneumocystis***,** we used a quantitative touchdown PCR method that targeted the multicopy MSG gene of *P. jirovecii* (*14*). In brief, primers JKK14/15 and JKK17 amplify a 250-bp segment of the multicopy MSG gene family. The MSG primers also amplify a 295-bp fragment of the artificially constructed internal control. Detection was carried out by using 2 separate sets of fluorescence resonance energy transfer (FRET) probes, which detected the MSG (PCMSGFRET1U and PCMSGFRET1D) and internal control target (PCMIM1U and PCMIM1D), respectively. The probes were labeled with Red640 and Red705, respectively, for simultaneous amplification and detection to take place in the same reaction tube. PCR conditions were as previously described (*14*). First, all samples were assayed with the internal control included. *P. jirovecii*–positive samples were then assayed for quantification without an internal control, including 2 standards (10^3^ copies/μL) in the experiment, and the generated external standard curve was imported for quantification.

For betaglobin, a commercial kit, Control Kit DNA (Roche), was used to estimate the amount of human DNA present in the samples. PCR conditions were as recommended by the company. Two standards (1.5 × 10^−1^ ng/μL) were included in each experiment, and the generated external standard curve was imported for quantification. All *P. jirovecii*–positive samples and a randomly selected subgroup of *P. jirovecii*–negative samples (all negative samples from patients born on the first through third days of the month) were assayed; 5 μL of patient specimen or the standard dilution was added per tube.

### Interpretation

If a PCR-positive sample was negative by the second analysis, the sample was reextracted and reassayed in 2 tubes. If at least 2 of 4 tubes were positive, the sample was recorded as positive for *P. jirovecii*.

A negative MSG result had to have a positive result for the internal control to be considered valid, to ensure absence of inhibitors in the specimen. If PCR inhibitors were detected, the sample was to be diluted 1:5.

### Data Analysis

All acquired fluorescence data were analyzed with LightCycler software (Roche). Clinical data were recorded with EpiData 2.1a (EpiData Association, Odense, Denmark). Statistics were calculated by using the SAS System, version 9.1 for Windows (SAS Institute Inc., Cary, NC, USA).

Wilcoxon 2-sample test or Kruskal-Wallis test was used to compare quantitative data when appropriate. Fisher exact test was used to compare groups. A 2-sided p value of <0.05 was considered significant. Values presented are medians with ranges or interquartile ranges (IQR). Logistic regression was used for univariate and multivariate analyses.

## Results

### Patients and Episodes

Four hundred sixty-one NPAs from 423 patients were available for analysis. One HIV-infected child with PCP was excluded from analysis. The remaining infants were presumed to be uninfected with HIV of the basis of review of their medical charts. Two hundred ninety-six (70%) patients (with 303 episodes [70%]) were hospitalized at Hvidovre University Hospital and the rest at Amager Hospital. Sixty-four percent of the episodes received a diagnosis of LRTI, 28% a diagnosis of URTI, and 8% “other.” The median age was 112 days (IQR 49–265), and 52.7% of the NPAs were from male patients.

### PCR Results

No samples exhibited inhibition. All controls were appropriate. Sixty-seven (16%) of the 422 patients had positive test results for *P. jirovecii* in 68 (16%) of 431 episodes. More than 1 NPA was collected in 21 episodes, of which 4 (19%) were *P. jirovecii* positive, and PCR results were concordant in 96% (46/48 samples) of the NPAs. NPAs from 8 pairs of siblings were collected, and all pairs were concordant (1/8 pairs positive).

Basic demographic data for the *P. jirovecii*–positive and –negative groups are presented in Table 1, and age distribution in quartiles is presented in Table 2. Significant differences were found in age, days admitted to hospital, and occurrence of reported fever. However, no significant difference was found in temperature at admission. No difference in positivity rate was seen between the 2 hospitals. Univariate and multivariate analyses are presented in Table 3. By univariate analysis, URTI versus LRTI, age quartiles 2 and 3 versus 1, and reported fever were associated with the presence of *P. jirovecii* but days admitted to the hospital was not. Age quartiles 2 and 3 versus quartile 1 and URTI versus LRTI were independently associated with *P. jirovecii* positivity by multivariate analysis. The distribution of number of episodes by clinical diagnosis and age is illustrated by Appendix Figure A and the frequency of *P. jirovecii*–positive episodes by Appendix Figure B.

**Table 1:** Basic demographic data for the *Pneumocystis jirovecii*–positive and *P. jirovecii*–negative groups

Demographic factor	No. *P. jirovecii* positive†	No. *P. jirovecii* negative*	p value
RSV† positive	30/68 (44)	165/363 (45)	0.93
Sex (male)	36/67 (54)	188/358 (53)	0.89
Coexisting conditions	8/67 (12)	65/358 (18)	0.29
Reported fever	35/64 (55)	247/351 (70)	0.02
Admission temperature (°C)	37.6 (37.0–37.9)	37.7 (37.1–38.5)	0.11
Age, d	90 (73–112)	140 (44–292)	0.04
Hospital days	2 (0–5)	3 (1–6)	0.04
Hospital (Hvidovre)	50/68 (74)	253/363 (70)	0.52

*Denominators are total number of episodes in each group. Values are numbers (%) or median (interquartile range). †RSV, respiratory syncytial virus.

**Table 2:** Quartiles of age in days with rate of RSV and *Pneumocystis jiroveci* positivity*

Age quartile	n	Age, d†	RSV positive, %	*P. jirovecii* positive, %
1	108	7–49 (32.5, [22.5–42])	48	2
2	105	50–112 (78, [63–96])	51	48
3	107	113–265 (173, [139–214])	44	13
4	105	268–4,430 (415, [319–542])	38	1

*RSV, respiratory syncytial virus. †Median in parentheses with interquartile range in brackets.

**Table 3:** Univariate and multivariate OR (95% CI) for *Pneumocystis jirovecii* positivity (logistic regression analysis)*

	OR univariate (95% CI)	OR multivariate (95% CI)†
LRTI	1	1
URTI (vs LRTI)	2.74 (1.58–4.73)	2.00 (1.05–3.82)
Other (vs LRTI)	0.70 (0.20–2.41)	1.06 (0.27–4.20)
Age Q1	1	1
Age Q2 vs Q1	48.2 (11.3–205)	47.4 (11.0–203)
Age Q3 vs Q1	7.98 (1.77–36.0)	8.74 (1.92–39.7)
Age Q4 vs Q1	0.51 (0.05–5.71)	0.60 (0.05–6.71)
Sex (M vs F)	1.05 (0.62–1.77)	
Coexisting conditions	0.61 (0.28–1.34)	
Reported fever	0.51 (0.30–0.88)	
Fever‡	0.86 (0.50–1.49)	
RSV	0.94 (0.56–1.58)	
Hospital days	0.94 (0.88–1.01)	

*OR, odds ratio; CI, confidence interval; LRTI, lower respiratory tract infection; URTI, upper respiratory tract infection; M, male; F, female; RSV, respiratory syncytial virus. †Including more variables in the model did not increase the fitness; including RSV showed it was not a confounder. ‡Defined by temperature at admission >37.5°C.

### Quantitative Analysis of PCR-positive Results

If >1 NPAs were collected during the same episode, average numbers of copies were calculated for that episode. The *P. jirovecii*–positive episodes had a median of 9 copies/tube (IQR 2.8–25).

Of the 387 *P. jirovecii*–negative NPAs, 49 (12.7%) were randomly selected for betaglobin analysis. The *P. jirovecii*–positive and –negative samples had a median of 129,400 (IQR 49,540–298,800) versus 95,410 (IQR 27,610–228,800) pg/tube, with no significant difference (p = 0.09).

Due to the natural variation of the specimens, *P. jirovecii* copy numbers were corrected for amount of human DNA in the sample (copies MSG per ng betaglobin). The PCR-positive episodes had a median of 0.069 (IQR 0.021–0.315) copies/ng betaglobin per tube.

The quantitative data were normally distributed when logarithm transformed (Figure). Quantitative data for age and clinical diagnosis subgroups are presented in Table 4. No significant differences were found among groups.

**Figure:**
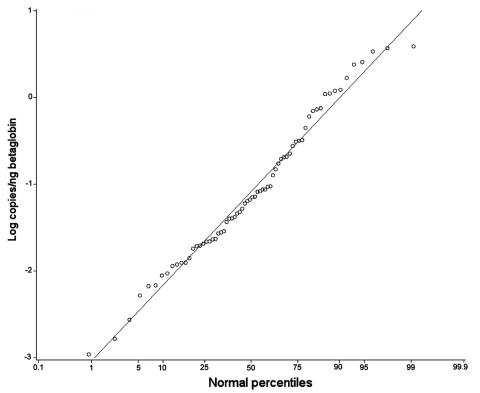
Plot of logarithmic transformed values of copies/ng betaglobin detected in PCR-positive tubes (○). Red line denotes normal distribution.

**Table 4:** Number of copies per ng of betaglobin detected per tube (median, IQR) in PCR-positive specimens in the 4 age groups and 3 diagnosis groups*†

	n	Median copies/ng betaglobin/tube (IQR)	p value
Age quartile			
1	2	0.854 (0.023–1.685)	0.37
2	50	0.063 (0.021–0.311)	
3	14	0.085 (0.022–0.318)	
4	1	0.001	
Clinical diagnosis			
LRTI	32	0.033 (0.016–0.269)	0.16
URTI	32	0.082 (0.033–0.526)	
Other	3	0.094 (0.087–1.098)	

*IQR, interquartile range; LRTI, lower respiratory tract infection; URTI, upper respiratory tract infection. †No significant differences were detected by Kruskal-Wallis tests.

## Discussion

In this study, *P. jirovecii* was detected in NPAs from 16% of infants hospitalized with acute RTI. A marked difference occurred in the age distribution, as the prevalence was 48% in infants ages 50 to 112 days (second quartile), 13% in infants ages 113 to 265 days (third quartile), and negligible in the youngest and oldest infants (Table 2, Appendix Figure). Similarly, ORs of 47 and 8.7 were found for the second and third quartiles, respectively, when compared with that of the youngest group for being *P. jirovecii* positive by multivariate analysis (Table 3). These data indicate that infants were exposed very early to *P. jirovecii*, and this raises the question of whether this diagnosis should be considered in infants ages 1.5–4 months who exhibit symptoms of an acute RTI.

The relative absence of *P. jirovecii* among the youngest infants (ages <50 days) could indicate either differences in exposure or in immunity, or reflect the incubation time of the infection. One could hypothesize that the increased rate of *P. jirovecii* positivity was coincidental with the infants’ introduction to a daycare facility/institution. However, the infants were cared for at home and not at an institution in 64 (96%) of the 67 *P. jirovecii–*positive episodes.

Another possible explanation is differences in immunity, which could be mediated by maternal antibodies in the youngest infants. Animal studies have shown that maternal antibodies are protective in infants (*15*–*17*). Likewise, *P. jirovecii* was seldom found in the oldest infants (>265 days of age), which may have been due to acquired immunity. Previous studies suggest that the clearance of organisms is complete; no detectable organisms were found by microscopy or PCR in postmortem lung specimens from immunocompetent adult patients (*18*). However, primary infection could possibly be acquired later in life and produce a milder illness (one that does not require hospitalization) in older children, and therefore these cases are not included in the current study.

Also, the absence of *P. jirovecii* among the youngest infants (ages <50 days) may have been a result of the incubation period of the infection, assuming that organism burden during the incubation period was below level of detection of the assay. Animal studies have indicated that the peak organism load in healthy mice occurs 5–6 weeks after exposure (*19*,*20*). Thus, the infants could have been exposed shortly after birth in order for symptoms to develop in infants at the ages found here. In fact, animal studies have found early exposure of newborn infants by a maternal source, and asymptomatic carriage by pregnant women has been reported recently (*21*–*23*). In a reported case of probable mother-to-infant transmission, the mother became symptomatic at 3 days postpartum and the infant at 29 days of age (*24*). The shorter incubation period in this case may reflect a higher level of infectious inoculum in this infant.

The age distribution was in concordance with the trend reported in a recent study on autopsied lung specimens from 112 infants (*25*). Similarly, serologic studies have indicated that most children seroconvert early in life (*2*–*5*,*8*,*26*). Among children with perinatally acquired HIV, the incidence of PCP was highest from 3 to 6 months of age (*27*,*28*). That is, these infants were slightly older when PCP was diagnosed. Assuming that they were exposed to *P. jirovecii* at the same time as healthy infants, the difference could be because a longer incubation time is needed for clinical PCP to develop in susceptible immunocompromised persons. Previous studies have reported an overall *P. jirovecii* prevalence of 25% (*8*), and 32% (*9*) in infants with acute RTI. When episodes were considered, however, the prevalence in the latter study was 17%, which was in concordance with the findings in our current study. The former study comprised 178 infants but did not include clinical data, and the latter study comprised a smaller population (74 infants with 178 episodes). Geographic variation or methodologic differences may account for the slight difference in reported prevalence. Our study used a single-round, closed-tube, PCR format for detection, which has a high sensitivity and a greatly reduced risk for carryover contamination (*14*). The difference in amount of human DNA detected in *P. jirovecii*–positive and –negative samples did not reach the 5% level of significance. If, in fact, a difference exists, this could be because sample quality varied, which means that we may have underestimated the true prevalence of *P. jirovecii* carriage. The difference could also have occurred because the presence of *P. jirovecii* increases the amount of, for example, inflammatory cells in the nasopharyngeal secretions (*29*), thereby increasing the amount of human DNA sampled. The current study confirms the previous reports that *P. jirovecii* can be detected in respiratory tract specimens from otherwise healthy infants with an acute RTI.

*Pneumocystis* is likely transmitted through the respiratory route (*30*). The reservoir for *P. jirovecii* is unknown but could include other persons or environmental sources, whereas animal reservoirs are unlikely because of the host specificity (*6*,*31*). Animal studies have shown that colonized mice may transmit the organism to immunosuppressed mice (*32*). Therefore, healthy children with a primary *P. jirovecii* infection may play a role in the circulation of the organism as previously suggested (*25*), although recent genotyping studies have yielded conflicting results (*10*,*33*).

*P. jirovecii*–positive episodes could represent either colonization or clinical overt disease. We have previously shown the assay used here provides reproducible quantitative results, and that a similar real-time quantitative PCR assay correlates well with the number of whole organisms in the sample (*14*,*34*). The fact that the quantitative data were normally distributed after logarithmic transformation (Figure), and that no differences in copy numbers were detected among groups (Table 4), may indicate that the *P. jirovecii*–positive episodes represent 1 biologic phenomenon.

To our knowledge, no previous evidence has shown a connection between clinical illness or specific symptoms and primary infection in children. It has been presumed to be an asymptomatic or mild, nonspecific disease (*6*,*22*). In the study by Vargas et al., no differences in clinical diagnosis were observed (*8*). In the current study, we found that infants with an episode of URTI were 2.0× more likely to be carrying *P. jirovecii* than infants with LRTI, when findings were adjusted for age (Appendix Figure, Table 3). This finding is somewhat surprising because the organism causes LRTI in immunocompromised subjects. It is unlikely that the finding is due to differences in sample quality, because the amount of betaglobin detected in samples from patients with URTI and LRTI was similar (data not shown), and no difference in adjusted *Pneumocystis* DNA was detected (Table 4). Parents reported that the child had a history of fever less often in *P. jirovecii*–positive episodes by univariate analysis, but no differences were found in the presence of fever as assessed at admission (Tables 1 and 3). Also, *P. jirovecii*–positive infants tended to be hospitalized for a marginally shorter duration (Tables 1 and 3).

The limitations of this study are primarily the lack of a healthy control group without respiratory symptoms, and lack of serologic data from the patients. Also, a comprehensive analysis of the specimens was not done for known respiratory pathogens other than RSV and *P. jirovecii*. Further investigation is therefore needed to confirm these findings before recommendations can be made for routine diagnostic testing for *Pneumocystis* in defined populations of infants, because it remains possible that *Pneumocystis* carriage in this population could represent a bystander phenomenon. Similarly, one should be cautious in inferring these results to infants without RTI or to those with an RTI that does not require hospitalization.

In this study, we found an overall prevalence of *P. jirovecii* in the respiratory tracts of 16% of infants hospitalized with an episode of acute RTI. Infants ages 50–112 days harbored *P. jirovecii* in 48% of the episodes. Our data suggest that primary *P. jirovecii* infection acquired early in life may present itself as a self-limiting URTI.

## Supplementary Material

Appendix Figure A) Total number of episodes grouped by clinical diagnosis and age. B) Percentage of Pneumocystis jirovecii-positive samples within subsets grouped by clinical diagnosis and age. *If total number of episodes <5, the bar has been removed.
