# Supplementary figures and images for: Primary Pneumocystis Infection in Infants Hospitalized with Acute Respiratory Tract Infection
Source: Emerg Infect Dis. 2007 Jan;13(1):66–72. doi: 10.3201/eid1301.060315 (PMC2725833; doi:10.3201/eid1301.060315)

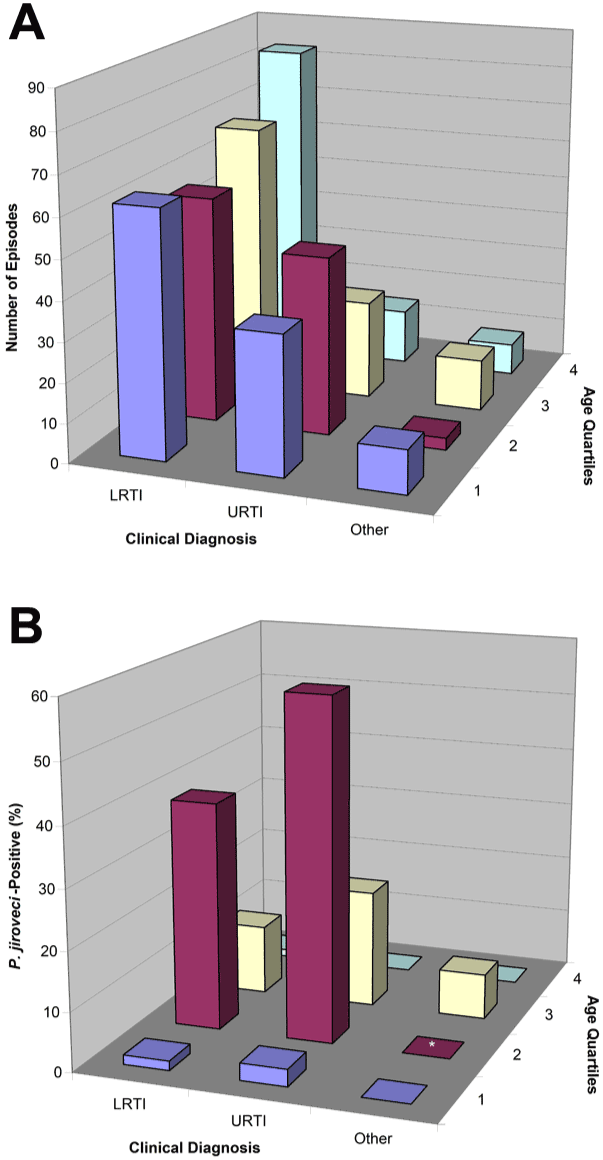

Supplement: Appendix Figure — A) Total number of episodes grouped by clinical diagnosis and age. B) Percentage of Pneumocystis jirovecii-positive samples within subsets grouped by clinical diagnosis and age. *If total number of episodes <5, the bar has been removed. [file 06-0315_app-s1.gif]
